# Supplementary material for: Analysis of Flavonoid Metabolites in Watercress (Nasturtium officinale R. Br.) and the Non-Heading Chinese Cabbage (Brassica rapa ssp. chinensis cv. Aijiaohuang) Using UHPLC-ESI-MS/MS
Source: Molecules. 2021 Sep 26;26(19):5825. doi: 10.3390/molecules26195825 (PMC8510128; doi:10.3390/molecules26195825)
Supplement: Supplementary file 1 [file molecules-26-05825-s001.zip › molecules-1334478-supplementary.pdf]

molecules

MDPI

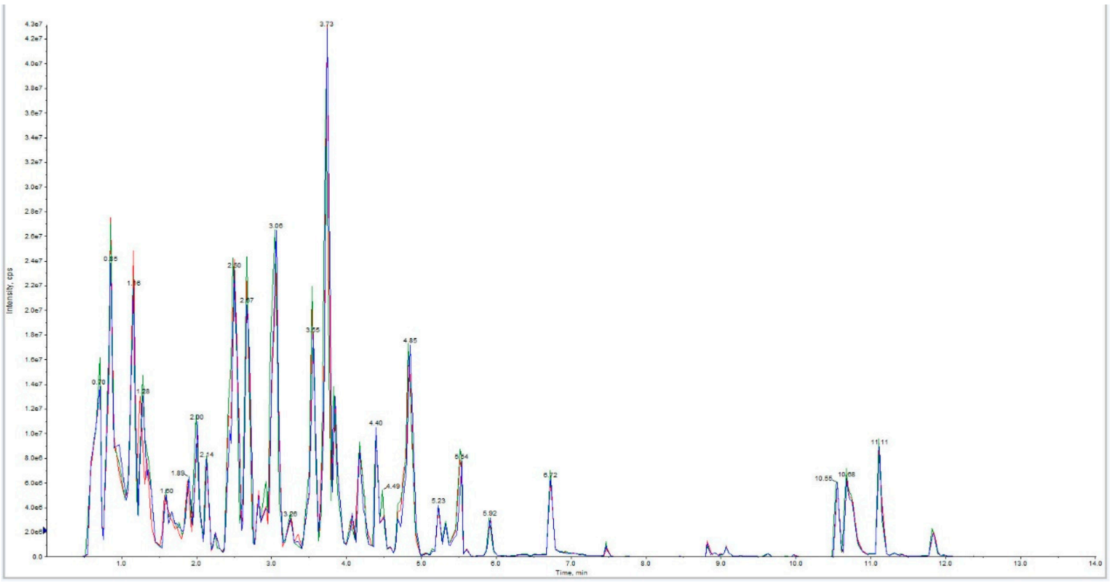

Figure S1. Total ions current overlaps of the three quality control samples by mass spectrometry detection.

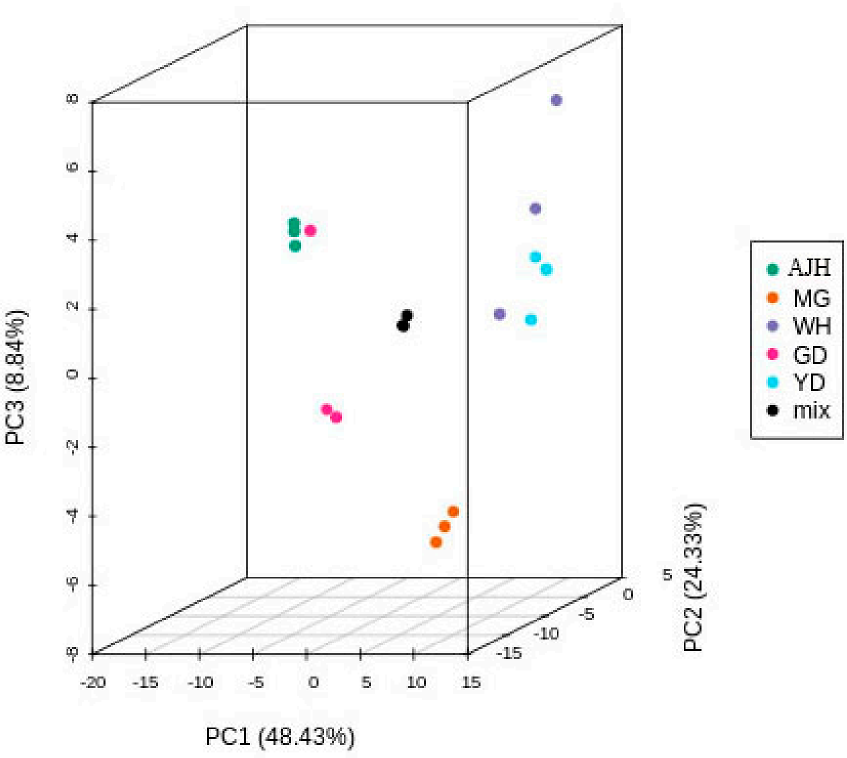

Figure S2. Differential flavonoid metabolite analysis on the basis of principal component (PCA) 3D plot.

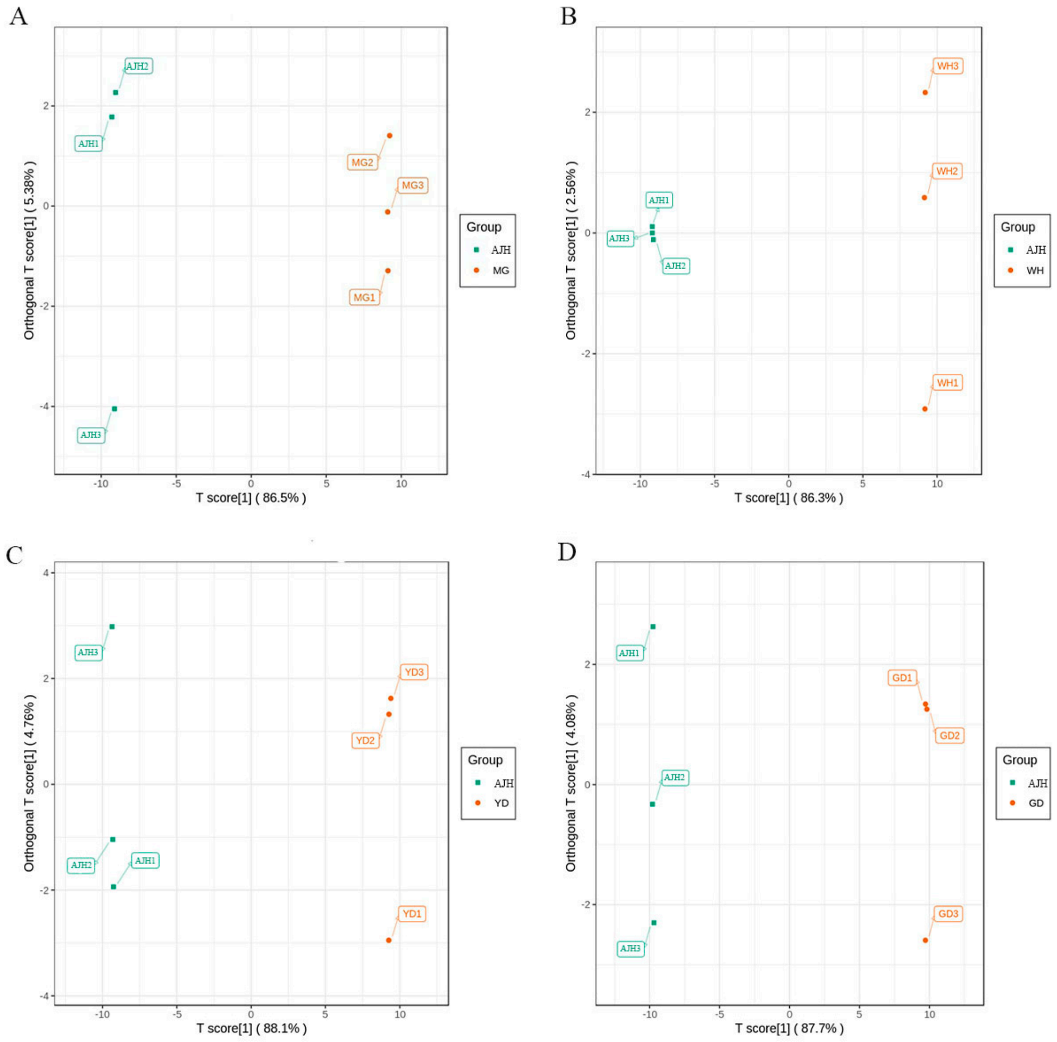

Figure S3. Summary of OPLS-DA model. The abscissa represents the predicted principal component; The ordinate represents the orthogonal principal component.

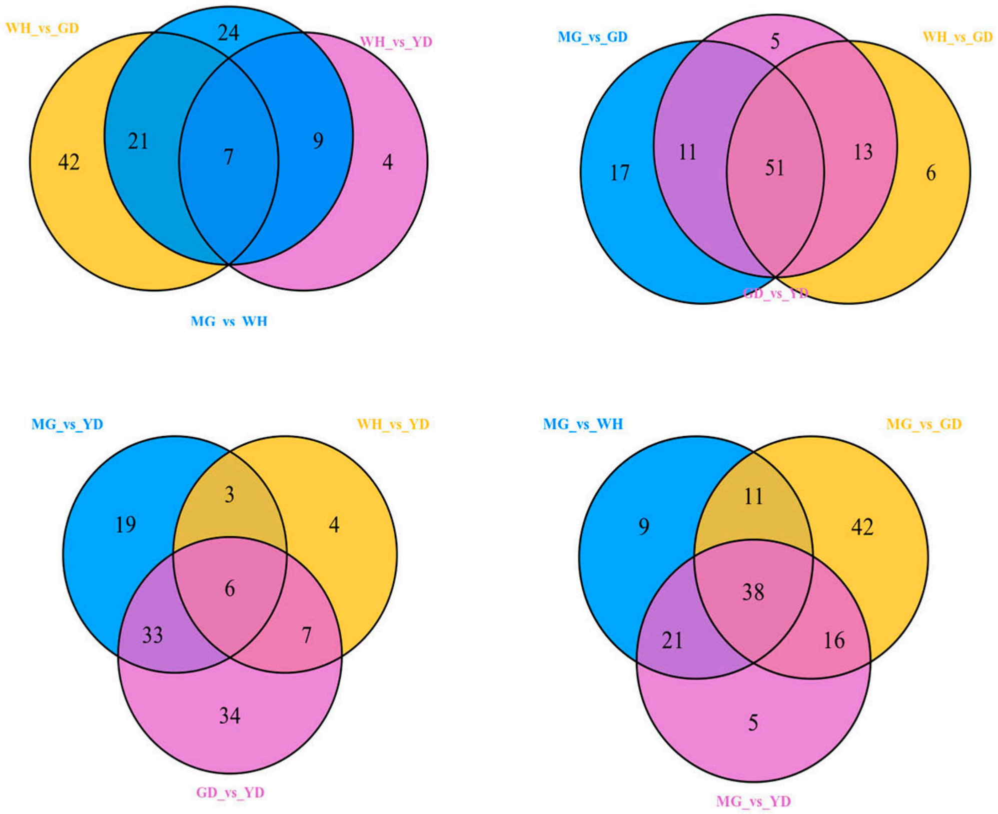

Figure S4. Venn diagram shows the overlapping and unique differential metabolites amongst the comparison groups.

Table S1. Information on standards and reagents.

Reagent	Purity	Manufacturers
Methanol	Chromatographic pure	Merck
Acetonitrile	Chromatographic pure	Merck
Standards	Chromatographic pure	BioBioPha/Sigma-Aldrich

Table S2. A list of the 132 metabolites detected in this study.

Index	Q1 (Da)	Q3 (Da)	Molecular Weight (Da)	Ionization Model	KEGG ID	Compounds	Class
pmb0550	4.49E+02	2.87E+02	4.49E+02	[M]+	C08604	Cyanidin-3-O-glucoside (Kuromanin)	Anthocyanins
pme1398	4.65E+02	3.03E+02	4.65E+02	[M]+	C12138	Delphinidin-3-O-glucoside (Mirtillin)	Anthocyanins
Zmcp002924	6.11E+02	2.87E+02	6.11E+02	[M]+	C16306	Cyanidin-3-O-(2''-O-glucosyl) glucoside	Anthocyanins
Zmcp002986	7.73E+02	2.87E+02	7.73E+02	[M]+	-	Cyanidin-3-O-sophoroside-5-O-glucoside	Anthocyanins
Zmcp002839	7.89E+02	3.03E+02	7.89E+02	[M]+	C16314	Delphinidin-3,5,3'-Tri-O-glucoside	Anthocyanins
Cwjp002051	9.19E+02	1.47E+02	9.19E+02	[M]+	-	Cyanidin-3-O-(6''-O-p-coumaroyl) sophoroside-7-O-glucoside	Anthocyanins
Zmcp004218	9.49E+02	2.87E+02	9.49E+02	[M]+	-	Cyanidin-3-O-(6''-O-feruloyl) sophoroside-5-O-Glucoside	Anthocyanins
Zmcp003437	9.79E+02	2.87E+02	9.79E+02	[M]+	-	Cyanidin-3-O-(6''-O-sinapoyl) sophoroside-5-O-glucoside	Anthocyanins
mws1179	4.33E+02	2.71E+02	4.34E+02	[M-H] -	C09099	Naringenin-7-O-glucoside (Prunin)*	Dihydroflavone
mws0046	5.79E+02	2.71E+02	5.80E+02	[M-H] -	C09789	Naringenin-7-O-Neohesperidoside (Naringin)	Dihydroflavone
mws0044	3.03E+02	1.25E+02	3.04E+02	[M-H] -	C01617	Dihydroquercetin(Taxifolin)	Dihydroflavonol
mws1174	3.13E+02	2.53E+02	3.14E+02	[M-H] -	C16418	3-O-Acetylpinobanksin	Dihydroflavonol
pme1598	4.63E+02	3.01E+02	4.64E+02	[M-H] -	--	Hesperetin-5-O-glucoside	Dihydroflavonol

pmp000004	3.31E+02	3.16E+02	3.30E+02	[M+H] +	--	4',5,7-Trihydroxy-3',6-dimethoxyflavone (Jaceosidin)	flavone
Lmzp004885	3.31E+02	3.15E+02	3.30E+02	[M+H] +	C10193	Tricin (5,7,4'-Trihydroxy-3',5'-dimethox-yflavone)	flavone
mws0043	4.03E+02	3.73E+02	4.02E+02	[M+H] +	C10112	Nobiletin (5,6,7,8,3',4'-Hexamethoxyfla-vone)	flavone
Lmbp003668	4.19E+02	2.87E+02	4.18E+02	[M+H] +	--	Kaempferol-3-O-arabinoside	flavone
HJN087	4.33E+02	2.71E+02	4.34E+02	[M-H] -	--	Naringenin-4'-O-glucoside*	flavone
pmn001697	4.45E+02	2.69E+02	4.46E+02	[M-H] -	--	Apigenin-7-O-glucuronide	flavone
pme2459	4.49E+02	2.87E+02	4.48E+02	[M+H] +	C03951	Luteolin-7-O-glucoside (Cynaroside)*	flavone
pmb3012	4.61E+02	2.99E+02	4.62E+02	[M-H] -	--	Chrysoeriol-7-O-glucoside*	flavone
Lmjp003655	4.63E+02	3.01E+02	4.62E+02	[M+H] +	--	6-C-Methyl Kaempferol-3-glucoside*	flavone
Lmjp003295	4.79E+02	3.17E+02	4.78E+02	[M+H] +	--	6-Methoxykaempferol-3-O-glucoside*	flavone
pmb0608	5.49E+02	3.01E+02	5.48E+02	[M+H] +	--	Chrysoeriol-7-O-(6''-malonyl) glucoside*	flavone
Zmhp006502	5.49E+02	3.01E+02	5.48E+02	[M+H] +	--	Kaempferide-3-O-(6''-malonyl) glucoside*	flavone
HJAP064	5.65E+02	3.17E+02	5.64E+02	[M+H] +	--	Isorhamnetin-3-O-(6''-malonyl) glucoside	flavone
HJAP148	5.81E+02	2.87E+02	5.80E+02	[M+H] +	--	Kaempferol-3-O-sambubioside	flavone
Lmsn002815	5.93E+02	2.85E+02	5.94E+02	[M-H] -	C21833	Kaempferol-3-O-rutinoside (Nicotiflorin)*	flavone
pmp001079	5.95E+02	4.49E+02	5.94E+02	[M+H] +	C12630	Luteolin-7-O-neohesperidoside (Lonic-erin)*	flavone
pmp000593	5.95E+02	4.49E+02	5.94E+02	[M+H] +	--	Luteolin-7-O-rutinoside*	flavone
Lmyp004022	6.01E+02	2.87E+02	6.00E+02	[M+H] +	--	Kaempferol-3-O-(2''-galloyl) glucoside	flavone
Hmpp002612	6.11E+02	2.87E+02	6.10E+02	[M+H] +	--	Luteolin-7-O-gentiobioside*	flavone
Zmxp003107	6.11E+02	2.87E+02	6.10E+02	[M+H] +	--	Luteolin-7,3'-di-O-glucoside*	flavone
Lmyn001269	6.09E+02	2.85E+02	6.10E+02	[M-H] -	C12634	Kaempferol-3-O-sophoroside*	flavone
HJN051	6.23E+02	3.15E+02	6.24E+02	[M-H] -	--	Tamarixetin-3-O-rutinoside*	flavone
mws1068	2.85E+02	1.51E+02	2.86E+02	[M-H] -	C05903	Kaempferol (3,5,7,4'-Tetrahydroxyflavone)	Flavonols
Lmcp004693	3.01E+02	2.58E+02	3.00E+02	[M+H] +	C10098	Kaempferide (3,5,7-Trihydroxy-4'-methox-yflavone)	Flavonols
mws0988	3.15E+02	1.65E+02	3.16E+02	[M-H] -	C10176	Rhamnetin*	Flavonols
mws0066	3.15E+02	1.51E+02	3.16E+02	[M-H] -	C10084	Isorhamnetin*	Flavonols
Lmdp003808	3.17E+02	3.02E+02	3.16E+02	[M+H] +	C10022	Azaleatin (5-O- Methylquercetin) *	Flavonols
mws0919	4.31E+02	2.85E+02	4.32E+02	[M-H] -	C16911	Kaempferol-3-O-rhamnoside (Afzelin)(Kaempferin)	Flavonols
pmp000117	4.33E+02	4.03E+02	4.32E+02	[M+H] +	--	3,5,6,7,8,3',4'-Heptamethoxyflavone	Flavonols
mws2186	4.35E+02	3.03E+02	4.34E+02	[M+H] +	--	Avicularin (Quercetin-3-O-α-L-arabino-furanoside) *	Flavonols
Lmdp003509	4.35E+02	3.03E+02	4.34E+02	[M+H] +	--	Quercetin-3-O-xyloside (Reynoutrin)*	Flavonols
mws2209	4.49E+02	2.87E+02	4.48E+02	[M+H] +	C12249	Kaempferol-3-O-glucoside (Astragalin)*	Flavonols
mws0913	4.47E+02	2.85E+02	4.48E+02	[M-H] -	C12626	Kaempferol-3-O-galactoside (Trifolin)*	Flavonols
Lmpn006208	4.61E+02	3.15E+02	4.62E+02	[M-H] -	--	8-Methoxykaempferol-7-O-rhamnoside	Flavonols
mws1329	4.63E+02	3.01E+02	4.64E+02	[M-H] -	C12639	Quercetin-7-O-glucoside*	Flavonols
mws0061	4.63E+02	3.00E+02	4.64E+02	[M-H] -	C10073	Quercetin-3-O-galactoside (Hyperin)*	Flavonols
Lmdp003286	4.65E+02	3.03E+02	4.64E+02	[M+H] +	--	Isohyperoside*	Flavonols
mws0091	4.63E+02	3.00E+02	4.64E+02	[M-H] -	C05623	Quercetin-3-O-glucoside (Isoquercitrin)*	Flavonols
Lmyp004444	4.79E+02	3.17E+02	4.78E+02	[M+H] +	--	Tricin-4'-methylether-3'-O-glucoside*	Flavonols
Hmcp002207	4.79E+02	3.17E+02	4.78E+02	[M+H] +	--	Isorhamnetin-7-O-glucoside (Brassicin)*	Flavonols
Lmmp003903	4.91E+02	2.87E+02	4.90E+02	[M+H] +	--	Kaempferol-3-O-(2''-acetyl) glucoside*	Flavonols
Lmmn003398	4.89E+02	2.85E+02	4.90E+02	[M-H] -	--	Kaempferol-3-O-(6''-acetyl) glucoside*	Flavonols
pmn001642	5.03E+02	4.59E+02	5.04E+02	[M-H] -	--	Kaempferol-3-O-(2''-O-acetyl) glucuronide	Flavonols

Zmsp004363	5.07E+02	3.03E+02	5.06E+02	[M+H] +	--	Quercetin-3-O-(6''-acetyl) glucoside*	Flavonols
Hmln002199	5.05E+02	3.00E+02	5.06E+02	[M-H] -	--	Quercetin-3-O-(6''-acetyl) galactoside*	Flavonols
pmn001644	5.19E+02	3.00E+02	5.20E+02	[M-H] -	--	Quercetin-3-O-(2''-acetyl) glucuronide	Flavonols
Li512111	5.19E+02	3.15E+02	5.20E+02	[M-H] -	--	Isorhamnetin-3-O-(6''-acetyl) glucoside	Flavonols
Lmdp004892	5.35E+02	2.87E+02	5.34E+02	[M+H] +	--	Kaempferol-3-O-(6''-malonyl) galactoside*	Flavonols
Lmmp003817	5.35E+02	2.87E+02	5.34E+02	[M+H] +	--	Kaempferol-3-O-(6''-malonyl) glucoside*	Flavonols
pmp000589	5.51E+02	3.03E+02	5.50E+02	[M+H] +	--	Quercetin-7-O-(6''-malonyl) glucoside*	Flavonols
Hmln002189	5.49E+02	5.05E+02	5.50E+02	[M-H] -	--	Quercetin-3-O-(6''-malonyl) galactoside*	Flavonols
mws1290	5.93E+02	2.85E+02	5.94E+02	[M-H] -	C17140	Kaempferol-3-O-(6''-p-coumaroyl) glucoside (Tiliroside)	Flavonols
pme1605	5.93E+02	2.85E+02	5.94E+02	[M-H] -	--	Kaempferol-3-O-robinobioside (Biorobin)*	Flavonols
Lmjp002867	5.95E+02	2.87E+02	5.94E+02	[M+H] +	--	Kaempferol-3-O-neohesperidoside*	Flavonols
Lmjp002596	5.97E+02	3.03E+02	5.96E+02	[M+H] +	C12637	Quercetin-3-O-xylosyl (1→2) glucoside*	Flavonols
Lmtp004044	5.97E+02	3.03E+02	5.96E+02	[M+H] +	--	Quercetin-3-O-apiosyl (1→2) galactoside*	Flavonols
Hmbp001825	5.97E+02	3.03E+02	5.96E+02	[M+H] +	--	Quercetin-3-O-sambubioside*	Flavonols
Lmjp002461	6.11E+02	3.03E+02	6.10E+02	[M+H] +	--	Quercetin-3-O-neohesperidoside*	Flavonols
mws0059	6.09E+02	3.01E+02	6.10E+02	[M-H] -	C05625	Quercetin-3-O-rutinoside (Rutin)*	Flavonols
pmb0711	6.11E+02	3.03E+02	6.10E+02	[M+H] +	--	Quercetin-7-O-rutinoside*	Flavonols
Lmmp003091	6.11E+02	3.03E+02	6.10E+02	[M+H] +	C17563	Quercetin-3-O-(4''-O-glucosyl) rhamnoside*	Flavonols
Lmbp002336	6.11E+02	3.03E+02	6.10E+02	[M+H] +	--	Quercetin-3-O-(2''-O-rhamnosyl) galactoside*	Flavonols
pmn001583	6.09E+02	3.00E+02	6.10E+02	[M-H] -	--	Quercetin-3-O-robinobioside*	Flavonols
Lmmp002963	6.25E+02	3.17E+02	6.24E+02	[M+H] +	--	6-C-Methylquercetin-3-O-rutinoside*	Flavonols
pmp001310	6.27E+02	3.03E+02	6.26E+02	[M+H] +	--	6-Hydroxykaempferol-3,6-O-Diglucoside*	Flavonols
pmp001311	6.27E+02	3.03E+02	6.26E+02	[M+H] +	--	6-Hydroxykaempferol-7,6-O-Diglucoside*	Flavonols
Lmtp003677	6.27E+02	3.03E+02	6.26E+02	[M+H] +	C12667	Quercetin-3-O-sophoroside (Baimaside)*	Flavonols
Hmcp001578	6.41E+02	4.79E+02	6.40E+02	[M+H] +	--	Isorhamnetin-3,7-O-diglucoside*	Flavonols
Hmcp001629	6.97E+02	4.49E+02	6.96E+02	[M+H] +	--	Kaempferol-3-O-(6''-Malonyl) glucoside-7-O-Glucoside	Flavonols
pmb0706	7.13E+02	4.65E+02	7.12E+02	[M+H] +	--	Quercetin-3-O-(6''-malonyl) glucosyl-5-O-glucoside*	Flavonols
Lmmp002995	7.13E+02	3.03E+02	7.12E+02	[M+H] +	--	Quercetin-7-O-(2''-malonyl) glucosyl-5-O-glucoside*	Flavonols
Hmcp001658	7.27E+02	4.79E+02	7.26E+02	[M+H] +	--	Isorhamnetin-3-O-(6''-malonylglucoside)-7-O-glucoside	Flavonols
mws1035	7.41E+02	4.33E+02	7.40E+02	[M+H] +	C10178	Kaempferol-3-O-robinoside-7-O-rhamnoside (Robinin)	Flavonols
HJAP061	7.43E+02	3.03E+02	7.42E+02	[M+H] +	--	Quercetin-3-O-(2''-O-arabinosyl) rutinoside	Flavonols
pmp001105	7.57E+02	2.87E+02	7.56E+02	[M+H] +	--	Kaempferol-3-O-neohesperidoside-7-O-glucoside*	Flavonols
Lmpp003268	7.57E+02	2.87E+02	7.56E+02	[M+H] +	--	Kaempferol-3-O-rutinoside-7-O-glucoside*	Flavonols
Hmcp001329	7.59E+02	3.03E+02	7.58E+02	[M+H] +	--	Quercetin-3-O-xylosyl (1→2) glucosyl (1→2) glucoside	Flavonols
Lmdp004336	7.73E+02	3.25E+02	7.72E+02	[M+H] +	--	Kaempferol-3-O-(6'''-Caffeoyl) glucosyl-(1→2)-Galactoside	Flavonols
Lmmp002334	7.73E+02	3.03E+02	7.72E+02	[M+H] +	--	Quercetin-3-O-rutinoside-7-O-glucoside*	Flavonols
Lmqp002170	7.73E+02	4.49E+02	7.72E+02	[M+H] +	C12635	Kaempferol-3-O-sophorotrioside*	Flavonols
Lmdp004668	7.87E+02	3.39E+02	7.86E+02	[M+H] +	--	Kaempferol-3-O-(6''-Feruloyl) glucosyl-(1→4)-galactoside	Flavonols
Lmdp004160	7.89E+02	3.25E+02	7.88E+02	[M+H] +	--	Quercetin-3-O-(2'''-Caffeoyl) sophoroside	Flavonols
pmp001312	7.89E+02	3.03E+02	7.88E+02	[M+H] +	--	6-Hydroxykaempferol-3,7,6-O-triglycoside	Flavonols
Lmdp004574	8.03E+02	3.39E+02	8.02E+02	[M+H] +	--	Quercetin-3-O-(2'''-Feruloyl) sophoroside	Flavonols
Lmdp004696	8.17E+02	3.69E+02	8.16E+02	[M+H] +	--	Kaempferol-3-O-(6''-Sinapyl) glucosyl-(1→2)-Galactoside	Flavonols

Lmdp004426	8.33E+02	3.69E+02	8.32E+02	[M+H] +	--	Quercetin-3-O-[2''-O-(6'''-sinapoyl) glucosyl] glucoside	Flavonols
Lmdp004516	8.59E+02	3.25E+02	8.58E+02	[M+H] +	--	Kaempferol-3-O-(2''-caffeoyl) glucosyl-(1→2) -(6''-malonyl) glucoside	Flavonols
Lmqp002349	8.59E+02	6.97E+02	8.58E+02	[M+H] +	--	Kaempferol-3-O-(6''''-malonyl) sophorotrioside	Flavonols
Lmdp005088	8.59E+02	3.69E+02	8.58E+02	[M+H] +	--	Kaempferol-3-O-(2''-sinapoyl) glucosyl-(1→2) -(6''-acetyl) glucoside	Flavonols
Lmdp004967	8.73E+02	3.39E+02	8.72E+02	[M+H] +	--	Kaempferol-3-O-(2''-feruloyl) glucosyl-(1→2)- (6''-malonyl) glucoside	Flavonols
Lmdp004221	8.75E+02	3.25E+02	8.74E+02	[M+H] +	--	Quercetin-3-O-(2''-Caffeoyl) glucoside-(1→2)- (6''-Malonyl) glucoside	Flavonols
Lmdp004638	8.89E+02	3.39E+02	8.88E+02	[M+H] +	--	Quercetin-3-O-(6''-feruloyl) glucoside-7-O-(6''-malonyl) glucoside	Flavonols
Lmdp004819	9.03E+02	3.69E+02	9.02E+02	[M+H] +	--	Kaempferol-3-O-(2''-sinapoyl) glucosyl-(1→2)- (6''-malonyl) glucoside	Flavonols
Lmwp005248	9.03E+02	2.87E+02	9.02E+02	[M+H] +	--	Kaempferol-3-O-(6'''-p-Coumaroyl) glucosyl-(1→2)-Glucoside-7-O-Rhamnoside	Flavonols
Lmdp004550	9.19E+02	3.69E+02	9.18E+02	[M+H] +	--	Quercetin-3-O-(6''-sinapoyl) glucoside-7-O-(6''-malonyl) glucoside	Flavonols
Cwjp002681	9.19E+02	2.87E+02	9.18E+02	[M+H] +	--	Kaempferol-3-O-(2'''-p-Coumaroyl) sophoroside-7-O-Glucoside	Flavonols
Lmdp004904	9.33E+02	3.69E+02	9.32E+02	[M+H] +	--	Kaempferol-3-O-neohesperidoside-7-O-(2''-feruloyl) glucoside	Flavonols
Lmdp004011	9.35E+02	3.25E+02	9.34E+02	[M+H] +	--	Quercetin-3-O-(6''-Caffeoyl) sophoroside-7-O-rhamnoside	Flavonols
Hmap001882	9.35E+02	2.87E+02	9.34E+02	[M+H] +	--	Kaempferol-3-O-sophorotrioside-7-O-glucoside	Flavonols
Cwjp002007	9.49E+02	1.77E+02	9.48E+02	[M+H] +	--	Kaempferol-3-O-sophoroside-7-O-(2''-Feruloyl) glucoside	Flavonols
Lmdp004461	9.49E+02	3.39E+02	9.48E+02	[M+H] +	--	Quercetin-3-O-(6''-feruloyl) glucoside-7-O-rutinoside	Flavonols
Hmap001789	9.51E+02	3.03E+02	9.50E+02	[M+H] +	--	Quercetin-3-O-gentiotetroside	Flavonols
Lmdp004580	9.63E+02	3.69E+02	9.62E+02	[M+H] +	--	Kaempferol-3-O-neohesperidoside-7-O-(2''-sinapoyl) glucoside	Flavonols
Lmdp004311	9.79E+02	3.69E+02	9.78E+02	[M+H] +	--	Quercetin-3-O-(6''-sinapoyl) glucoside-7-O-rutinoside	Flavonols
Lmtp002474	5.65E+02	4.27E+02	5.64E+02	[M+H] +	--	Apigenin-6-C-(2''-glucosyl) arabinoside*	Flavonoid carbonoside
Lmnp102580	5.65E+02	4.33E+02	5.64E+02	[M+H] +	--	Apigenin-6-C-(2''-xylosyl) glucoside*	Flavonoid carbonoside
Lmyp004081	5.79E+02	3.13E+02	5.78E+02	[M+H] +	--	Isovitexin-2''-O-rhamnoside*	Flavonoid carbonoside
Lmlp002990	5.95E+02	3.13E+02	5.94E+02	[M+H] +	--	Isosaponarin (Isovitexin-4'-O-glucoside)	Flavonoid carbonoside
pmb0665	6.11E+02	4.65E+02	6.10E+02	[M+H] +	--	Luteolin-8-C-glucoside-7-O-glucoside*	Flavonoid carbonoside
pmb0624	6.11E+02	4.31E+02	6.10E+02	[M+H] +	--	Luteolin-6-C-glucoside-7-O-glucoside*	Flavonoid carbonoside
pmp000126	6.11E+02	5.93E+02	6.10E+02	[M+H] +	C10102	Luteolin-6,8-di-C-glucoside	Flavonoid carbonoside
pmb0660	7.57E+02	1.47E+02	7.56E+02	[M+H] +	--	Luteolin-6-C-glucoside-7-O-(6''-p-coumaroyl) glucoside	Flavonoid carbonoside
pmb0639	7.57E+02	5.95E+02	7.56E+02	[M+H] +	--	Apigenin-8-C-glucoside-7-O-Sophoroside	Flavonoid carbonoside
pmb0662	7.87E+02	4.63E+02	7.86E+02	[M+H] +	--	Luteolin-6-C-glucoside-7-O-(6''-feruloyl) glucoside	Flavonoid carbonoside
pmb0666	8.01E+02	6.39E+02	8.00E+02	[M+H] +	--	Apigenin-6-C-glucoside-7-O-(6''-sinapoyl) glucoside	Flavonoid carbonoside
pma0791	5.21E+02	2.73E+02	5.20E+02	[M+H] +	--	Naringenin-7-O-(6''-malonyl) glucoside	Flavanols
Lmgp004731	4.33E+02	2.71E+02	4.32E+02	[M+H] +	--	Genistein-7-O-galactoside*	Isoflavones
Lmhp003217	6.25E+02	3.17E+02	6.24E+02	[M+H] +	--	2'-Hydoxy,5-methoxyGenistein-O-rhamnosyl-glucoside*	Isoflavones
Lmhp002800	6.41E+02	3.17E+02	6.40E+02	[M+H] +	--	2'-Hydoxy,5-methoxyGenistein-4',7-O-diglucoside*	Isoflavones

The asterisk stands for indistinguishable isomers.
